# The Relationship between Serum Lipids and Sudden Sensorineural Hearing Loss: A Systematic Review and Meta-Analysis

**DOI:** 10.1371/journal.pone.0121025

**Published:** 2015-04-13

**Authors:** I Jen Chang, Chung Jan Kang, Chen Yu Yueh, Ku Hao Fang, Re Ming Yeh, Yao Te Tsai

**Affiliations:** 1 Department of Family Medicine, Chang Gung Memorial Hospital, Chiayi, Taiwan; 2 Department of Otolaryngology-Head and Neck Surgery, Chang Gung Memorial Hospital, Taoyuan, Taiwan; 3 Department of Otolaryngology-Head and Neck Surgery, Chang Gung Memorial Hospital, Chiayi, Taiwan; IRCCS Istituto Oncologico Giovanni Paolo II, ITALY

## Abstract

**Background:**

Sudden sensorineural hearing loss (SSNHL) is a relatively common condition that is usually of unknown etiology. A number of individual studies have investigated the association between various serum lipids and SSNHL; however, the findings have been inconsistent. In an attempt to obtain more definitive information on the relationship between serum lipids and SSNHL, we carried out a systematic review and meta-analysis.

**Methods:**

Medline, the Cochrane Library, and EMBASE were searched using the following key words: lipid, cholesterol, triglyceride, fat, serum, blood, sudden hearing loss, hearing loss, hearing disorders. Randomized controlled trials, prospective cohort studies, and retrospective case-control studies involving patients with SSNHL and healthy controls that examined the relationship (reported as odds ratios [OR]) between lipid profiles and SSNHL were included. Primary outcomes were total cholesterol and low-density lipoprotein cholesterol (LDL-C) concentrations. Secondary outcomes were triglyceride, high-density lipoprotein cholesterol, and lipoprotein(a) concentrations.

**Results:**

A total of 6 case-control studies were included in this systematic review/meta-analysis. The total number of participants ranged from 30 to 250 in the case group and from 43 to 271 in the control group. Meta-analysis revealed no significant difference in total cholesterol levels between the case and control groups (pooled OR = 1.79, 95% confidence interval [CI] = 0.98 to 3.26, *P* = 0.057). Likewise, meta-analysis revealed no significant difference in LDL-C concentrations between the case and control groups (pooled OR = 1.15, 95% CI = 0.64 to 2.07, *P* = 0.639). Since there were an insufficient number of studies reporting data for the secondary outcomes, meta-analysis was not possible.

**Conclusions:**

Our results do not provide evidence for serum lipids being associated with SSNHL, nor do they definitively rule out such an association. Additional studies are needed to ascertain the relationship, or lack thereof, between serum lipids and SSNHL.

## Introduction

Sudden sensorineural hearing loss (SSNHL), or sudden deafness, is typically defined as the rapid hearing loss of at least 30 dB in 3 contiguous audiometric frequencies within 3 days [1]. The incidence of SSNHL has been estimated to range from 5 to 20 cases per 100,000 in general population and occurs most commonly among adults aged in their 50s and 60s [2,3]. The onset of SSNHL, typically first noticed on waking in the morning, may be sudden or develop over several days [4], and is unilateral in the vast majority of cases [2]. In addition to hearing loss, individuals with SSNHL may experience a range of other bothersome symptoms, including tinnitus, vertigo, and sensation of ear fullness [5].

Sudden sensorineural hearing loss is considered to be a medical emergency [5], and as such, requires prompt evaluation/treatment. Treatment for SSNHL is dependent on the underlying cause (if identifiable); in the case of idiopathic SSNHL whose cause is not known, a short course of corticosteroids is often prescribed [5,6]. A variety of factors are known to affect recovery from SSNHL, including the duration and severity of hearing loss, age, and the presence of other symptoms [6]. But impaired hearing has also been reported to gradually return to normal without any medical intervention in up to 65% of patients with idiopathic SSNHL [2,7]. So time to recovery in patients with hearing loss and a definitive underlying pathology may depend on the underlying cause(s) [6], but is otherwise difficult to gauge for patients with hearing loss of unknown etiology.

In most instances (approximately 70% of all cases), SSNHL is idiopathic in origin [4], and various etiologic mechanisms have also been proposed for SSNHL. These can be broadly classified as infectious, otologic, traumatic, vascular, and neoplastic, and have been reviewed in detail elsewhere [4–6]. There is some evidence that dyslipidemia may be a risk factor for SSNHL [8–11], and has been hypothesized to contribute to the initiation of an inflammatory or stressful response in the inner ear, leading to SSNHL [11]. There is also evidence that lipid-lowering therapy (specifically low-density lipoprotein cholesterol [LDL-C] apheresis) can be more effective than traditional treatment for patients with SSNHL who have elevated serum LDL-C concentrations [11]. But there is still no consensus as there are findings from other studies which do not support this assertion that dyslipidemia is a risk factor for SSNHL [12,13].

Given the aforementioned lack of definitive information from individual studies, we carried out a systematic review and meta-analysis of the literature examining the relationship between serum lipids and SSNHL.

## Materials and Methods

### Selection criteria

Randomized controlled trials, prospective cohort studies, and retrospective case-control studies were considered for inclusion if they involved patients with SSNHL and healthy controls, and examined the relationship (determined as odds ratios [ORs]) between lipid profiles and SSNHL. Prospective cohort studies in which assessments were made before and after the onset of SSNHL were also considered for inclusion.

Studies published in the form of letters, comments, editorials, or case reports were excluded. Non-English language publications were also excluded.

### Search strategy

Medline, the Cochrane Library, and EMBASE were searched on 14 April 2014 using combinations of the following key words: lipid, cholesterol, triglyceride, fat, serum, blood, sudden hearing loss, hearing loss, hearing disorders.

Reference lists of pertinent studies were also hand-searched to identify other potentially relevant studies not retrieved in the literature search.

### Study selection and data extraction

Studies were identified by two independent reviewers using the search strategy already described. A third reviewer was consulted to resolve any disagreements between the two primary reviewers.

The following information and/or data were extracted from studies that met the eligibility criteria: name of the first author, year of publication, study design, number of participants in each treatment group, participants’ age and gender, serum cholesterol, triglyceride, LDL-C, high-density lipoprotein C, and lipoprotein(a) concentrations, and associations (ORs) between lipid parameters and SSNHL.

### Quality assessment

The Newcastle-Ottawa Quality Assessment Scale was used to assess quality of the studies included in the systematic review/meta-analysis, and was performed by two reviewers with a third reviewer consulted in case of discrepancy. All studies received a score of 7 or 8, indicating good qualities.

### Outcome measures

Comparisons were made between patients with SSNHL (case group) and control patients (control group). The primary outcomes were total cholesterol and LDL-C concentrations. The secondary outcomes were concentrations of triglyceride, HDL-C, and lipoprotein(a).

### Statistical analysis

Pooled ORs were calculated for the primary outcomes (case vs control). Heterogeneity among the studies was assessed using the Cochran Q and the I^2^ statistic. For the Q statistic, *P* <0.10 indicates statistically significant heterogeneity. For the I^2^ statistic, which indicates the percentage of the observed between-study variability that is due to heterogeneity and not to chance, 0 to 25% indicates no heterogeneity, 25% to 50% indicates moderate heterogeneity, 50% to 75% indicates large heterogeneity, and 75% to 100% indicates extreme heterogeneity. If either Q statistic (*P* <0.1) or I^2^ statistic (>50%) indicated the existence of heterogeneity between studies, a random-effects model (DerSimonian–Laird method) of analysis would be used; otherwise, a fixed-effects model (Mantel-Haenszel method) would be used instead. A two-sided *P* value <0.05 was considered to be statistically significant. All statistical analyses were performed using the statistical software Comprehensive Meta-Analysis, version 2.0 (Biostat, Englewood, NJ).

## Results

### Literature search

**Fig 1:**
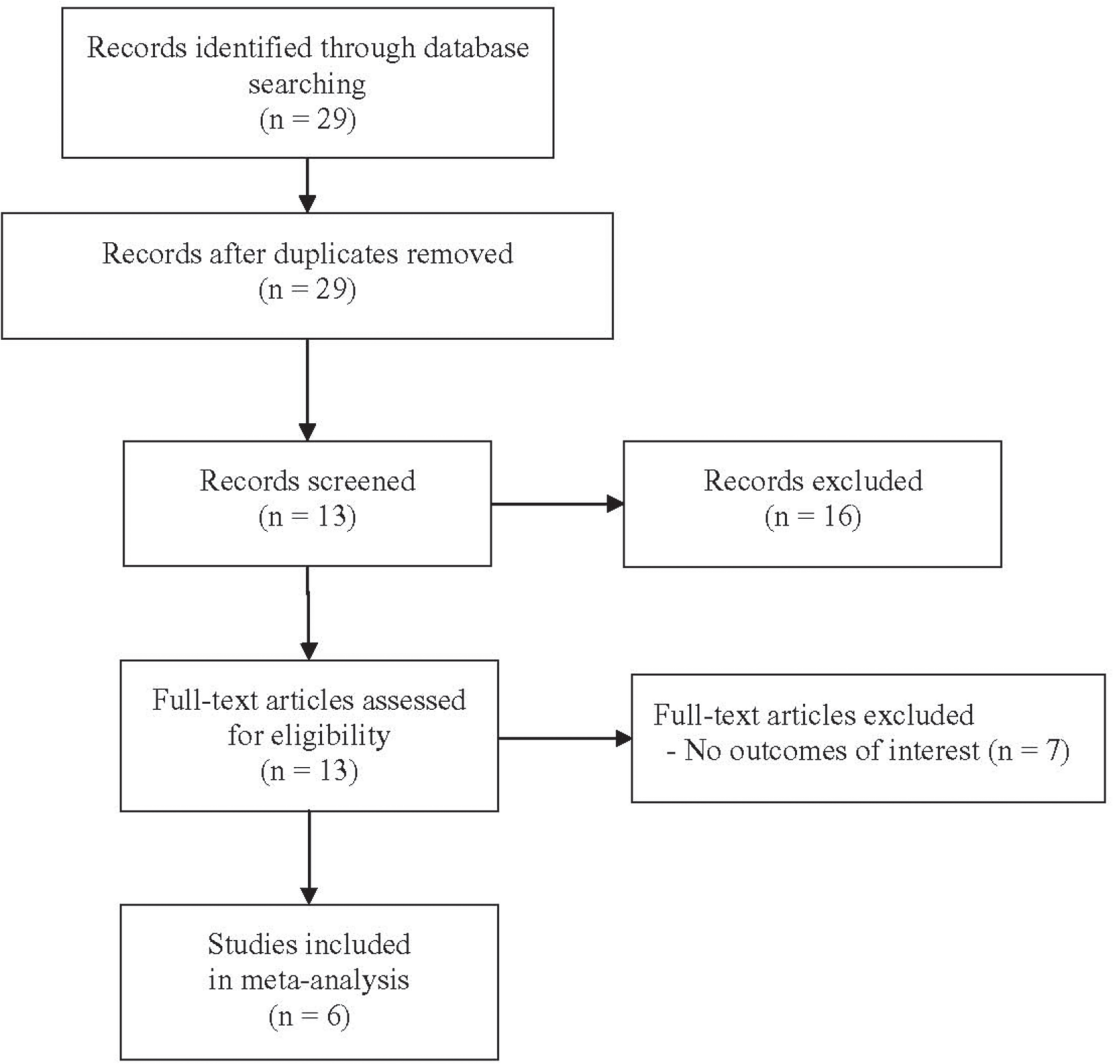
Flow chart of study selection.

A total of 29 unique articles were identified in the literature search (Fig 1). Of these, 16 did not meet the eligibility criteria on title/abstract review and were excluded. The remaining 13 articles underwent full-text review. Among those 13 articles, 7 [14–20] were subsequently excluded because they did not report outcomes of interest (specifically, the values of odd ratio and/or 95% confidence limits), so only 6 studies [9–12,21,22] were eligible for inclusion in the meta-analysis.

### Study characteristics

All of the 6 studies included in the meta-analysis were case-control studies, of which the key characteristics are summarized in Table 1. The total number of participants ranged from 30 to 250 in the case group and from 43 to 271 in the control group. Participants were generally around 50 years of age, with the mean age ranging from 45.5 to 56 years in the case group and from 43 to 55 years in the control group. There was a slightly higher proportion of male participants in 4 of the 6 studies [9,11,12,22]. Overall, the proportion of males in the studies ranged from 43.3% to 54.8% in the case group and from 43.2% to 60.5% in the control group. In 4 of the 5 studies [9–11,22], total cholesterol concentrations were higher in the case group (range: 200 to 227 mg/dL) than in the control group (range: 175 to 227 mg/dL). Likewise, in 3 of the 4 studies [10,11,22], LDL-C concentrations were higher in the case group (range: 114 to 131 mg/dL) than in the control group (range: 111 to 124 mg/dL). Only 2 studies each reported participants’ triglyceride [9,22], HDL-C [11,22], and lipoprotein(a) [21,22] concentrations. Triglyceride concentrations ranged from 117.0 to 124.4 mg/dL in the case group and from 128.2 to 128.4 mg/dL in the control group. HDL-C concentrations ranged from 55.3 to 61 mg/dL in the case group and from 54 to 56.3 mg/dL in the control group. Lipoprotein(a) concentrations were reported as mean (standard deviation) in 1 study [22] and median (range) in the other [21] study.

**Table 1:** Summary of basic characteristics of studies included in meta-analysis.

Author(Year)	Comparison	Subjects (N)	Age^†^ (years)	Male(Year)	TC^†^(mg/dL)	TG†(mg/dL)	LDL-C^†^(mg/dL)	HDL-C^†^ (mg/dL)	LP(a)^†^ (mg/L)
Weng (2013)	Case	250	56.41	54.8	182.9 (39.4)	117.0 (85.6)	103.4 (33.1)	55.3 (15.5)	5434 (5151)
	Control	250	(15, 84)^#^		169.0 (36.2)	128.2 (287.5)	91.9 (27.9)	56.3 (16.2)	6201 (7285)
Aimoni (2010)	Case	141	54.6	53.2	227.2 (40.0)	124.4 (64.9)	NA	NA	NA
			(15.8)						
	Control	271	55.0	52.4	214.4 (40.8)	128.4 (101.9)	NA	NA	NA
			(15.8)						
Cadoni (2010)	Case	43	50.0	44.2	213 (44)	NA	131 (32.40)	NA	NA
			(14)						
	Control	43	43	60.5	175 (21.43)	NA	110.82 (22.66)	NA	NA
			(11)						
Cadoni (2007)	Case	30	45.5	43.3	200 (38.95)	NA	128 (35.89)	NA	NA
			(23, 72)^#^						
	Control	60	49.5	43.3	175 (26.51)	NA	110.7 (31.34)	NA	NA
			(23, 77)^#^						
Rudack (2006)	Case	142	51.2	54.2	215 (32)	NA	114 (29)	61 (16)	NA
			(17.2)						
	Control	84	49.8	54.2	227 (38)	NA	124 (29)	54 (14)	NA
			(13.6)						
Marcucci (2005)	Case	155	54	43.2	NA	NA	NA	NA	111
			(19, 79 ^#^						(1, 1146)*
	Control	155	54	43.2	NA	NA	NA	NA	103
			(19, 78)^#^						(11, 695)*

Data expressed as:

^†^ mean (standard deviation)

^#^ mean (range)

* median (range)

Abbreviations: TC, total cholesterol; TG, triglyceride; LDL-C, low density lipoprotein cholesterol; HDL-C, high density lipoprotein cholesterol; LP(a), Lipoprotein(a); NA, no data available.

### Assessment of serum lipids as risk factors for SSNHL

Table 2 summarizes the findings from the studies included in the meta-analysis regarding the assessment of serum lipids as risk factors for SSNHL. All but 1 study [22] provided results for multivariate regression analyses in the determination of ORs. All 6 studies reported serum total cholesterol as a risk factor for SSNHL. Various cut-off concentrations were used in this assessment both within and between studies; however, higher concentrations of total cholesterol were associated with an increased risk of SSNHL in 5 of 6 studies [9–11,21,22]. The strength of the relationship was highly variable between studies. Four studies [10–12,22] reported serum LDL-C as a risk factor for SSNHL, with all the findings showing that the risk of SSNHL was decreased with lower LDL-C concentrations. Three studies [9,21,22] reported serum triglycerides as a risk factor for SSNHL. The results varied between studies and depending on the cut-off concentrations used. Only 2 studies each reported serum HDL-C and lipoprotein(a) concentrations as risk factors for SSNHL; the findings were inconsistent between these studies.

**Table 2:** Summary of the assessment of serum lipids as risk factors (determined as odds ratios) for sudden sensorineural hearing loss for studies included in meta-analysis.

			Odds Ratio		
Author(Year)	TC	TG	LDL-C	HDL-C	LP(a)
Weng (2013)	1.459 (1.211–1.758)^c^	0.976 (0.897–1.062)^c^	1.628 (1.287, 2.061)^c^	0.847 (0.552, 1.300)^c^	0.999 (0.999–1.000)^c^
Aimoni (2010)	>235 vs ≤200:	>140 vs ≤86:	NA	NA	NA
	2.25 (1.25, 4.03)^a^; 201–235 vs ≤200: 1.87 (1.07, 3.24)^a^	1.19 (0.67, 2.11)^a^;			
		87–139 vs ≤86:			
		1.00 (0.58, 1.74)^a^			
Cadoni (2010)	>200 vs ≤200:	NA	<130 vs >a130:	NA	NA
	33.76 (1.96, 562.31)^a^		0.74 (0.02, 25.41)^a^		
Cadoni (2007)	>200 vs ≤200:	NA	<130 vs >130:	NA	NA
	36.7 (3.25, 414.52)^a^		0.17 (0.01, 1.98)^a^		
Rudack (2006)	0.780 (0.433, 1.405)^c^	NA	0.934 (0.519, 1.680)	1.937 (1.068, 3.513)^c^	NA
Marcucci (2005)	>190 vs <147:	>98 vs <82:	NA	NA	>163 vs <67:
	19 (7, 50.1)^a^;	3.0 (0.3, 42)^a^;			1.8 (0.5, 4.1)^a^;
	148–190 vs <147: 4.8 (1.9, 12.6)^a^	82–98 vs <82:			68–163 vs <67:
		0.6 (0.4, 1.3)^a^			1.2 (0.5, 2.53)^a^

Abbreviations: TC, total cholesterol; TG, triglyceride; LDL-C, low-density lipoprotein cholesterol; HDL-C, high-density lipoprotein cholesterol; LP(a), lipoprotein(a); NA, no data available.

^a^, adjusted odds ratio (95% confidence interval)

^c^, crude odds ratio (95% confidence interval);

### Meta-analysis

Meta-analysis was performed for the total cholesterol and LDL-C concentrations. As only 2 studies reported useable results for triglyceride, HDL-C, and lipoprotein(a) concentrations, meta-analysis was not performed for these variables.

### Total cholesterol

**Fig 2:**
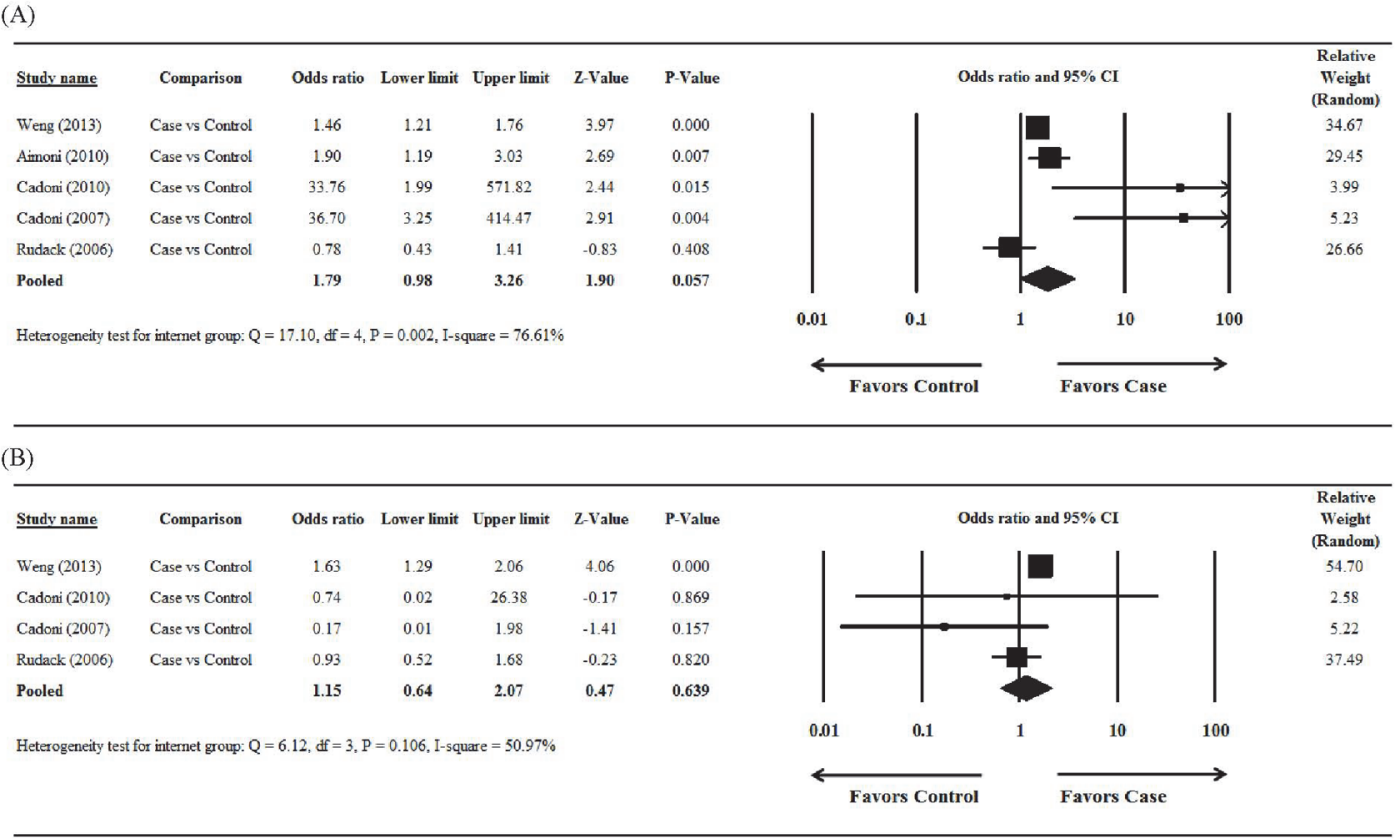
Forest plots showing the results of the meta-analysis of (A) total cholesterol and (B) low-density lipoprotein cholesterol for the case/sudden sensorineural hearing loss group vs the control group. Abbreviation: CI, confidence interval.

The study by Marcucci et al [21] did not report useable data and was excluded from this analysis. There was significant heterogeneity when data from the 5 studies were pooled (Q = 17.10, df = 4, *P* = 0.002, I^2^ = 76.61%); therefore, a random-effects model of analysis was used (Fig 2A). The overall analysis revealed that there was no significant difference in total cholesterol levels between the case and control groups (pooled OR = 1.79, 95% confidence interval [CI] = 0.98 to 3.26, *P* = 0.057).

### Low-density lipoprotein cholesterol

The studies reported by Aimoni et al [9] and Marcucci et al [21] did not report useable data and were excluded from this analysis. There was significant heterogeneity when data from the 4 studies were pooled (Q = 6.12, df = 3, *P* = 0.106, I^2^ = 50.97%); therefore, a random-effects model of analysis was used (Fig 2B). The overall analysis revealed that there was no significant difference in LDL-C concentrations between the case and control groups (pooled OR = 1.15, 95% CI = 0.64 to 2.07, *P* = 0.639).

### Sensitivity analysis

**Fig 3:**
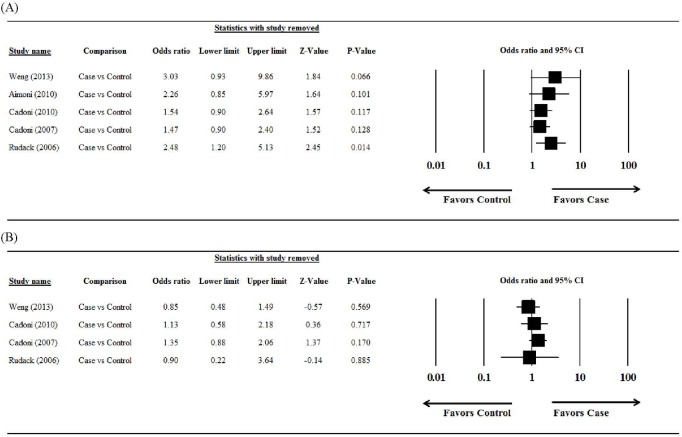
Results of the sensitivity analysis examining the influence of individual studies on pooled estimates, as determined using the leave-one-out approach, for (A) total cholesterol and (B) low-density lipoprotein cholesterol (case/sudden sensorineural hearing loss group vs control group). Abbreviation: CI, confidence interval.

The results of the sensitivity analysis (leave-one-out approach to) are summarized in Fig 2. For total cholesterol, the pooled estimate was different when the leave-one-out approach was used, indicating that the meta-analysis had poor reliability (Fig 3A). For LDL-C, the direction and magnitude of pooled estimates did not vary considerably when the leave-one-out approach was used, indicating that the meta-analysis had good reliability (Fig 3B).

### Publication bias

Publication bias could not be assessed for the meta-analysis outcomes because there were fewer than the minimum of 5 studies required to detect funnel plot asymmetry [23].

## Discussion

In this systematic review and meta-analysis, we examined the relationship between serum lipid concentrations and SSNHL. A total of 6 case-control studies met the criteria for inclusion in our study. Overall, our results do not support a relationship between serum lipids and SSNHL. However, given the small number of studies included and other limitations in this systematic review and meta-analysis, it is reasonable to assume our results are less than definitive and further research is warranted.

Meta-analysis was performed for two outcomes assessed in our study, the concentrations of total cholesterol and LDL-C. In both instances, there was no significant difference between the case (SSNHL) and control groups, suggesting that these variables are not associated with SSNHL. Interestingly, most studies individually reported that total cholesterol concentrations were higher in patients with SSNHL compared with control patients. Our findings suggest that factors other than SSNHL such as diet, lifestyle, and/or comorbidities may account for the higher concentrations of total cholesterol in the individual studies. There was considerable variability among the individual studies concerning the relationship between total serum cholesterol concentrations and SSNHL. Cadoni et al [10,11] reported a remarkably strong association relative to all other studies, but their studies included relatively small numbers of participants and thus carried less weight in our meta-analysis. The reasons underlying the striking findings reported by Cadoni et al may reflect different participant characteristics and/or factors accounted for in the analyses.

In addition to the concentrations of serum total cholesterol and LDL-C, we also retrieved information on several secondary outcomes, including the concentrations of serum triglyceride, HDL-C, and lipoprotein(a). Unfortunately, there were few data available for these outcomes so we were unable to perform meta-analysis for them. As a result, we cannot draw any conclusion on the relationship between these other types of lipids and SSNHL.

A number of studies [14–16,18,19] were excluded from our analysis for not providing outcomes of interest (the values of odd ratios) after full-text review, but they do deserve some discussion. Findings from these studies were inconsistent, with some suggesting that there may be an association between serum lipids and SSNHL, and others disputing such an association. Specifically, Lu et al [14] found that the concentrations of total cholesterol, triglyceride, and lipoprotein(a) were significantly higher in patients with SSNHL compared with controls, while Oreskovic et al [19] reported only higher concentrations of cholesterol and LDL-C were found in patients with SSNHL. Chang et al [16] suggested that hypercholesterolemia may be an independent risk factor for SSNHL. In contrast, Mosnier et al [15] found no difference in prevalence of hyperlipidemia between patients with SSNHL and controls, while Ullrich et al [18] reported that patients with SSNHL had serum lipid profiles similar to those of normal individuals. These studies are a reflection of broader coverage of this topic in literature, showing a lack of consensus concerning the relationship between serum lipids and SSNHL. One previous systematic review and meta-analysis on SSNHL in adults examined the association of acquired and inherited cardiovascular risk factors, including medical history (hypertension, diabetes mellitus, stroke and ischemic cardiopathy) and life style habits such as smoking and alcohol consumption, with SSNHL [24]. Interestingly, the authors found that history of smoking and alcohol consumption were associated with an increased risk of developing SSNHL, but hypertension and diabetes mellitus were not. However, they did not include dyslipidemia in their analysis, and our study filled this void. Given that dyslipidemia is also an established risk factor for cardiovascular disease, a link between dyslipidemia and SSNHL would not be unexpected.

Our systematic review and meta-analysis has a number of limitations that should be acknowledged. First, only a small number of studies were eligible for inclusion, which reduced the power of our meta-analysis. Second, as already mentioned, there were insufficient data available to perform meta-analysis for the association between SSNHL and concentrations of other lipids such as triglyceride, HDL-C and lipoprotein(a). Third, sensitivity analysis for total cholesterol indicated that the meta-analysis for this variable had poor reliability (reflecting the considerable variability between 2 of the studies included and the remaining studies). Hence, these results must be interpreted with caution. Finally, because of the small number of studies found to be eligible for inclusion in the meta-analyses, we were not able to perform an assessment of publication bias.

In summary, the results of our systematic review and meta-analysis do not provide sufficient evidence for the association between serum lipids and SSNHL, and the information currently available in the literature on this subject is by no means definitive. Hence, additional studies are warranted to fully ascertain the relationship between serum lipids and SSNHL, in order to enhance our understanding of this enigmatic condition.

## Supporting Information

S1 PRISMA Checklist PRISMA 2009 Checklist. (DOC)

S1 File Supplementary materials. (DOC)
