# Supplementary material for: The Relationship between Serum Lipids and Sudden Sensorineural Hearing Loss: A Systematic Review and Meta-Analysis
Source: PLoS One. 2015 Apr 13;10(4):e0121025. doi: 10.1371/journal.pone.0121025 (PMC4395088; doi:10.1371/journal.pone.0121025)
Supplement: S1 File — (DOC) [file pone.0121025.s002.doc]

**Supplementary materials**

*List of full-text articles excluded for lacking outcome(s) of interest (specifically, for not providing numerical values of odd ratio (OR) and/or 95% confidence limits for analysis)*

1. Ullrich D, Aurbach G, Drobik C. A prospective study of hyperlipidemia as a pathogenic factor in sudden hearing loss. Eur Arch Otorhinolaryngol. 1992;249(5):273-6.
2. Orita S, Fukushima K, Orita Y, Nishizaki K. Sudden hearing impairment combined with diabetes mellitus or hyperlipidemia. Eur Arch Otorhinolaryngol. 2007 Apr;264(4):359-62.
3. Lu YY, Jin Z, Tong BS, Yang JM, Liu YH, Duan M. A clinical study of microcirculatory disturbance in Chinese patients with sudden deafness. Acta Otolaryngol. 2008 Nov;128(11):1168-72.
4. Nagaoka J, Anjos MF, Takata TT, Chaim RM, Barros F, Penido Nde O. Idiopathic sudden sensorineural hearing loss: evolution in the presence of hypertension, diabetes mellitus and dyslipidemias. Braz J Otorhinolaryngol. 2010 May-Jun;76(3):363-9.
5. Mosnier I, Stepanian A, Baron G, Bodenez C, Robier A, Meyer B, Fraysse B, Bertholon P, Defay F, Ameziane N, Ferrary E, Sterkers O, de Prost D. Cardiovascular and thromboembolic risk factors in idiopathic sudden sensorineural hearing loss: a case-control study. Audiol Neurootol. 2011;16(1):55-66.
6. Oreskovic Z, Shejbal D, Bicanic G, Kekic B. Influence of lipoproteins and fibrinogen on pathogenesis of sudden sensorineural hearing loss. J Laryngol Otol. 2011 Mar;125(3):258-61.
7. Chang SL, Hsieh CC, Tseng KS, Weng SF, Lin YS. Hypercholesterolemia is correlated with an increased risk of idiopathic sudden sensorineural hearing loss: a historical prospective cohort study. Ear Hear. 2014 Mar-Apr;35(2):256-61.
